# Acknowledgement of Reviewers

**Published:** 2025-01-01

Dear Readers,

Our reviewers perform a very important and precious role in the evaluation of scientific articles and make valuable contributions to the increasing quality. Editorial Board would like to thank all the reviewers listed below for their support in the Turkish Journal of Gastroenterology in 2024:

Abdulbari Bener

Abdullah Buyruk

Abdullah Yeniova

Ahmet Tezel

Ahmet Cevdet Ceylan

Ahmet Eminler

Ahmet Sedat Boyacıoğlu

Ahmet Tekin

Ahmet Uygun

Aleksandra Klisic

Alessandro Granito

Ali Atay

Ali Bal

Ali Hatemi

Ali Şahin Küçükaslan

Alina Tantau

Alp Atasoy

Alpaslan Tanoğlu

Altay İldan

Altay Kandemir

Altay Kandemir

Andrea Lisotti

Anil Delik

Anil A. Chuturgoon

Antonio Facciorusso

Arif Aslaner

Arif Coşar

Armağan Günal

Arzu Tiftikçi

Asuman Karhan

Audrius Dulskas

Ayça Arzu Sayıner

Ayfer Karlitepe

Ayhan Hilmi Çekin

Ayşe Kefeli

Aysel Ünlüsoy Aksu

Bahadır Batar

Banu Kara

Batuhan Başpınar

Bayram Toraman

Begüm Özturk

Begümhan Ömeroğlu Gülada

Bekir Kocazeybek

Bengi Ruken Yavuz

Berat Ebik

Berna Tezcan Yavuz

Bilal Ergul

Bilal Toka

Bilger Çavuş

Brian Ting

Buğra Tolga Konduk

Bülent Baran

Burak Özşeker

Burçak Kayhan

Burcu Güven

Burçin Kaymaz

Burçin Pehlivanoğlu

Bürge Ulukan

Byong Duk Ye

Çağdaş Aktan

Çağlayan Keklikkiran

Canan Alkım

Canberk Tomruk

Carlo Catassi

Catalina Mihai

Cem Simsek

Cemile Merve Seymen

Ceyda Tuna Kirsaclıoğlu

Chiyi He

Chung-Ming Chen

Cihan Agalar

Cihan Kaya

Cihangir Akyol

Coşkun Demirtaş

Çağdaş Erdoğan

Çağdaş Kalkan

Çağla Kayabaşı

Derya Arı

Derya Okuyan

Dhiraj Agarwal

Didem Torun

Dilara Turan Gökçe

Dilek Cevik

Dilek Oğuz

Diler Taş Kılıç

Dimitry S. Bordin

Duygu Aygüneş

Duygu Bircan Kadıoğlu

Duygu Eryavuz Onmaz

Ebru Bilget Güven

Eda Balkan

Eda Kaya

Elham Bahador Zirh

Elif Bahadır

Elif Tuncel

Engin Altıntaş

Enqiang Linghu

Enver Avcı

Erdem Akbal

Erdem Koçak

Erkan Parlak

Erkin Öztaş

Erman Mercan

Ewan Glassey

Fatih Avsar

Fatih Güzelbulut

Fatih Kıvrakoğlu

Fatma Akın

Fatma Alibaz Öner

Fehmi Ateş

Ferdane Sapmaz

Figen Özcay

Filiz Akyüz

Fulya Gunsar

Genco Gençdal

Giovanni Sisti

Giuseppe Losurdo

Gladson Bertolini

Gökçe Tuncer Süral

Göksel Bengi

Gözde Dervis Hakim

Gupse Adali

Gürkan Celebi

Gülen Lied

Gülşah Seydaoğlu

Gürhan Şişman

Hailin Tang

Hakan Ünal

Hakan Çamyar

Hakan Yildiz

Hakan Yirgin

Hale Güler Kara

Hao Chi

Haojie Huang

Hasan Eruzun

Hasan Yilmaz

Hiroshi Kishikawa

Hüseyin Arikan

Hüseyin Ataseven

Hüseyin Korkmaz

Hüseyin Köseoğlu

Ilgın Yıldırım Şimşir

Istemi Serin

İbrahim Gecili

İbrahim Hatemi

İdris Kurt

İlkay Ergenç

İlkay İdilman

İlyas Tenlik

İrfan Koruk

İsmail Kalkan

Jinluan Li

Joo Young Cho

Kader Irak

Kamil Özdil

Kazım Yalçın Arğa

Kenan Moral

Konstantinos Makrilakis

Kubilay Doğan Kılıç

Kübra Akan

Kübra Narcı

Kyung-Goo Lee

Levent Erdem

Li Cui

Lizhi Lv

Maria Elisa Drago-Serrano

Marten A. Lantinga

Mehdi Koushki

Mehmet Orman

Mehmet Yalnız

Meral Yüksel

Mesut Sezikli

Metin Çalışkan

Metin Yalçın

Muhammer Ergenç

Muhammet Akpınar

Muharrem Keskin

Muhsin Muhip Murat Harputluoğlu

Müjde Soytürk

Mukaddes Tozlu

Murat Akyıldız

Murat Çakır

Murat Kahramaner

Murat Kekilli

Murat Özbayoğlu

Murat Saruç

Murat Törüner

Mustafa Arslan

Mustafa Can Güler

Mustafa Cengiz

Mustafa Gül

Mustafa Kaplan

Mustafa Tahtacı

Mücahit Seçme

Müge Öçal Demirtaş

Müjdat Zeybel

Mzamo Mbelle

Nalan Ünal

Nathaniel Cohen

Nazım Demircan

Nergiz Ekmen

Neşe Imeryüz

Neşe Atabey

Nihal Gökman

Nilay Danış

Nurcan Baykam

Onur Keskin

Orhan Aras

Orhan Coşkun

Orhan Sezgin

Osman Aydin

Osman Köstek

Oytun Erbaş

Ömer Binicier

Öykü Yürekli

Özdal Ersoy

Özge Saatçi

Özlem Mutluay Soyer

Özlem Tuğçe Çilingir-Kaya

Ömer Öztürk

Özlem Gül

Özlem Ünal Uzun

Pelin Balçik Erçin

Rafael Romero-Castro

Raim İliaz

Ramazan Dertli

Rasim Eren Cankurtaran

Richard Taubert

Sabite Kacar

Sahar A. El-Masry

Sahar Ravanshad

Salih Tokmak

Sami Fidan

Samy Azer

Seçkin Özgül

Sedef Göçmen

Sedef Kuran

Selcan Cesur

Selim Öğüt

Selman Alkan

Serdar Akça

Serdar Oter

Serkan Ocal

Serkan Torun

Serkan Yaraş

Şeyma Ünsal Beyge

Sezen Güntekin Ergün

Sezgi Kıpcak

Sezgin Barutçu

Sezgin Vatansever

Sözen Erdi

Şule Ayla

Sulen Sarıoğlu

Süleyman Günay

Suna Yapalı

Sushma Agrawal

Şahika Cıngır Köker

Şencan Acar

Şeymanur Yılmaz Taşçı

Tarkan Karakan

Tavga Aziz

Thania Kahn

Tolga Düzenli

Tuba Mutlu

Tufan Egeli

Tuğba Başoğlu

Tuğçe Eşkazan

Tuna Önal

Tutku Soyer

Uday C. Ghoshal

Ufuk Kutluana

Umit Karaoğullarından

Vishal Sharma

Wen-Xie Xu

Xiaoliang Wu

Xingming Jiang

Yahya Atayan

Yanli Zhu

Yaprak Dönmez Çakıl

Yekbun Adıgüzel

Yeliz Cagan Appak

Yılmaz Bilgic

Yongfang Yuan

Yoshiyasu Kono

Yucel Yankol

Zehra Kaya

Zehra Pakoz

Zekeriya Düzgün

Zeren Barış

Zeynep Gok Sargin

Zhendong Jin

Zhengjie Wang

Zhigang Zhang

Zhongjun Wu

